# Complete mitochondrial genome analysis of *Phascolosoma* sp. (Sipuncula, Phascolosomatida, Phascolosomatidea) from Micronesia

**DOI:** 10.1080/23802359.2017.1325337

**Published:** 2017-05-12

**Authors:** Jin-Mo Sung, Mustafa Zafer Karagozlu, Chang-Bae Kim

**Affiliations:** Department of Bioengineering, Sangmyung University, Seoul, Korea

**Keywords:** Sipuncula, Phascolosomatida, Phascolosomatidea, mitochondrial genome, *Phascolosoma* sp

## Abstract

In this study a Sipuncula species *Phascolosoma* sp. was collected from seagrass area from Chuuk lagoon Micronesia and its complete mitochondrial genome analyzed. This is the second complete mitochondrial genome record from the genus after *Phascolosoma esculenta*. The total length of mitochondrial genome of the species is 16,571 bp, which is longer than *P. esculenta* record. Also, locations of tRNA-Gly and putative control region are different between two records. Furthermore, phylogenetic relationship of *Phascolosoma* sp. are investigated due to protein-coding genes of mitochondrial genome. Due to the lack of recorded data, *P. esculenta* has been observed is the closest species to *Phascolosoma* sp. and they are belonging to the monophyletic group.

The genus *Phascolosoma* is one of the most species-rich genera in the phylum Sipuncula (Saiz et al. 2014) with the 25 valid species (WoRMS Editorial Board 2017). Almost all of these species are found in shallow water of the world’s oceans and inhabit cavities in solid substrates (Schulze et al. 2005). Although the many species are available, there is only one mitochondrial genome recorded from the genus which is *Phascolosoma esculenta* collected from China (Shen et al. 2009). In this study, a new complete mitochondrial genome from the genus *Phscolosoma* sp. is reported.

Specimens of *Phscolosoma* sp. have been collected from the sea grass area of Weno Island, Chook Lagoon, Federated States of Micronesia (7°26′41.0″N 151°53′57.7″E) on February 2015. The specimen genus has been identified morphologically and the collected specimen has been deposited in the Department of Life Science, Sangmyung University, with accession number SMU000510. Mitochondrial DNA sequencing, analysing, and phylogeny reconstruction methods were described in our previous study (Karagozlu et al. 2016).

Mitochondrial genome size of *Phscolosoma* sp. is evaluated as 16,571 bp long (GenBank accession number KX814447) and it is composed of 13 protein-coding genes, 2 ribosomal RNA genes, and 23 tRNA genes with base composition of 27.6% A, 22.4% C, 15.5% G, and 33.5% T. There are two overlapping regions in the genome and both are 1 bp lengths. Also, 31 intergenic sequences showed length variation ranging from 1 to 1116 bp. The gene order of the genome shows some difference from to that of *P. esculenta.* The main differences between structures of complete mitochondrial genomes are locations of tRNA-Gly and putative control region. The putative control region is located between tRNA-Glu and tRNA-Val with 459 bp length in the mitogenome of *Phascolosoma* sp. The same region is located between tRNA-Leu and NAD1 genes with 585 bp length in the mitogenome of *P. esculenta.* On the other hand, tRNA-Gly is located between ATP8 and tRNA-Gln genes in the mitogenome of *Phscolosoma* sp. while it located between tRNA-Val and ATP8 genes in the *P. esculenta* record. Also, there is tRNA-Arg in the mitogenome of *Phscolosoma* sp. which located between ATP6 and tRNA-His genes. This gene was not record in the *P. esculenta* mitogenome.

Phylogenetic relationship of *Phscolosoma* sp. in Trochozoa is investigated (Figure 1) in this study. The Trochozoa are composed of Sipuncula, Polychaeta, Mollusca, Annelida, Nemertea, and Brachiopoda phyla (Hejnol 2010). Due to reconstructed phylogenetic tree, *P. esculenta* is the closest species to *Phscolosoma* sp. and they belong to monophyletic group. The monophyly of the genus was previously well supported by nuclear and mitochondrial-based study (Kawauchi et al. 2012). The phylogenetic relationships of the Trochozoans due to mitochondrial genome-based phylogenetic study were supported by several molecular phylogenetic studies (Hausdorf et al. 2007; Witek et al. 2009; Hausdorf et al. 2010). Besides, there is a sister group relationship between Sipunculidea and Phascolosomatidea classes. The data show the similarity with previous transcriptomic-based study (Lemer et al. 2015). This genome data will be a part of mitochondrial genome library to provide evolutionary and systematic studies for the phylum Sipuncula.

**Figure 1.**
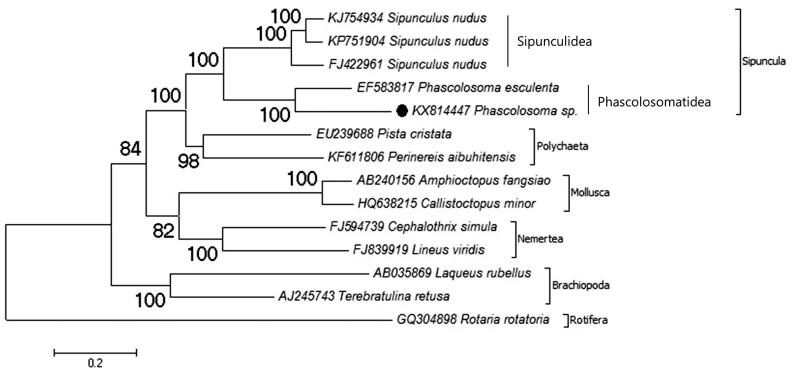
Phylogenetic relationship of *Phascolosoma* sp. (KX814447) in the Trochozoa. For reconstruction of the phylogenetic tree, two species from the every Trochozoa phylum selected and a Rotifera species selected as the out group. The mitochondrial genome of the selected species retrieved from the GenBank. The presented record marked with a dot.
